# Hospital-Based Injury Patterns Among Motorcycle Couriers and Pedestrians Struck by Courier-Operated Motorcycles and Mopeds: A Multicenter Retrospective Study

**DOI:** 10.3390/jcm15145383

**Published:** 2026-07-09

**Authors:** Yasin Köker, İsmet Teoman Benli, Fatih Şentürk, Muhammed Emin Yorulmaz, Turgut Akgül, Doğaç Karagüven, Ebubekir Bektaş, Tolga Onay, Sertaç Meydaneri, Murat Korkmaz, Funda Salgur

**Affiliations:** 1 Department of Orthopedics and Traumatology, Faculty of Medicine, Ufuk University, 06510 Ankara, Türkiye; 2 Department of Orthopedics and Traumatology, İstanbul Kanuni Sultan Süleyman Training and Research Hospital, 34303 İstanbul, Türkiye; 3 Department of Orthopedics and Traumatology, İstanbul Faculty of Medicine, İstanbul University, 34093 İstanbul, Türkiye; 4 Department of Orthopedics and Traumatology, Bolu İzzet Baysal State Hospital, 14300 Bolu, Türkiye; 5 Department of Orthopedics and Traumatology, Faculty of Medicine, İstanbul Medeniyet University, 34700 İstanbul, Türkiye; 6 Department of Orthopedics and Traumatology, Faculty of Medicine, Maltepe University, 34857 İstanbul, Türkiye; 7 Department of Family Medicine, Faculty of Medicine, Başkent University, Ankara Hospital, 06490 Ankara, Türkiye

**Keywords:** courier traffic accident, motorcycle courier, pedestrian injury, fracture, soft-tissue injury, surgical treatment, New Injury Severity Score

## Abstract

**Background and Objectives**: Courier-operated motorcycles and mopeds have become increasingly visible in urban traffic, raising concerns about injuries among both couriers and pedestrians struck by courier-operated vehicles. The study period coincided with the COVID-19 pandemic; however, this study was not designed to evaluate the effect of the pandemic on courier-related injuries. This multicenter retrospective study aimed to describe hospital-based injury patterns, injury severity, treatment requirements, complications, and mortality among selected courier-related casualties, including motorcycle couriers and pedestrians struck by courier-operated motorcycles or mopeds. **Materials and Methods**: This retrospective multicenter observational study included courier-related traffic casualties identified between 1 March 2020, and 1 June 2022, through nine hospitals in İstanbul and Ankara and, for fatal courier cases, linked emergency medical service and hospital records. The primary clinical cohort consisted of injured couriers and pedestrians evaluated or treated at participating hospitals. Linked prehospital and early fatal courier cases were analyzed separately for mortality-related and mechanism-specific fatality analyses. Injury severity was assessed using the New Injury Severity Score. Treatment was classified as conservative or surgical, including both fracture fixation and soft-tissue procedures. **Results**: A total of 857 courier-related traffic casualties were identified, including 491 couriers and 366 pedestrians. Among couriers, 111 fatal cases were verified through linked records. Clinical treatment and follow-up analyses were restricted to 380 hospital-treated surviving couriers and 366 pedestrians with available clinical records. In this selected hospital-treated courier cohort, multiple fractures, open fractures, higher injury severity scores, surgical treatment, and complications were more frequent than among pedestrians. All hospital-treated surviving couriers included in the orthopedic trauma cohort underwent surgery, reflecting the orthopedic trauma-based case-identification process, whereas most pedestrians were treated conservatively. **Conclusions**: Among this selected hospital-based cohort, courier casualties identified through hospital, trauma referral, emergency, forensic, and linked fatal-event records showed a more severe clinical profile than pedestrians evaluated after being struck by courier-operated motorcycles or mopeds. These findings should be interpreted as hospital-based injury-severity patterns rather than population-level estimates of accident incidence, relative injury risk, or public health burden.

## 1. Introduction

Motorcycle and moped couriers have become increasingly visible in urban traffic, particularly during periods of increased demand for delivery services. The study period coincided with the COVID-19 pandemic, during which delivery services became more prominent in daily urban mobility. However, the present study was not designed to evaluate the direct effect of the pandemic on courier-related injuries because it did not include pre-pandemic or post-pandemic comparison groups, temporal trend analysis, or exposure data.

Previous motorcycle trauma studies have generally evaluated motorcycle crashes as broad road-safety events, with emphasis on crash configuration, injury severity, helmet use, risky driving behavior, and demographic risk factors [1,2,3,4,5,6,7,8,9,10]. Courier-related crashes may represent a distinct clinical and occupational exposure pattern because couriers often ride in dense urban traffic and may be exposed to delivery-related time pressure, long working hours, and repeated traffic interactions [3,4,5,9,11]. Pedestrians struck by courier-operated vehicles represent an additional urban safety concern, but their injury spectrum may differ from that of couriers themselves.

This study aimed to describe injury patterns, injury severity, treatment requirements, complications, and mortality among selected courier-related casualties evaluated or identified through participating hospitals and linked fatal-event records. The study included motorcycle or moped couriers injured during delivery-related travel and pedestrians struck by courier-operated motorcycles or mopeds. Mechanisms of injury were characterized for both groups based on patient or relative accounts and available accident reports. Because the study was based on selected hospital-treated cases and linked fatal-event records, it was not designed to estimate population-level accident incidence, relative injury risk, or comparative public health burden between couriers and pedestrians.

## 2. Materials and Methods

### 2.1. Study Design, Setting, and Case Definition

This multicenter retrospective observational study included courier-related traffic casualties identified between 1 March 2020, and June 2022 through the medical records of nine participating hospitals in İstanbul and Ankara and, for fatal courier cases, linked official accident, emergency medical service, or forensic/police reports when available. The primary clinical cohort consisted of injured couriers and pedestrians who were evaluated or treated at the participating hospitals. Prehospital deaths were analyzed separately as a fatal-event subgroup and were not included in analyses of surgical treatment, complications, or follow-up outcomes.

Courier injuries were defined as injuries sustained by motorcycle or moped couriers during delivery-related travel. Pedestrian injuries were defined as injuries sustained by pedestrians struck by courier-operated motorcycles or mopeds. Because the study was based on hospital-treated or hospital-evaluated casualties, the findings describe injury-severity patterns among included cases and should not be interpreted as population-level accident risk estimates. The participating hospitals were trauma referral centers, and the courier cohort was derived mainly from hospital records, orthopedic trauma consultations, referral pathways, and linked fatal-event records. Therefore, minor courier injuries that did not require orthopedic evaluation, referral, or hospital-level treatment may not have been completely captured.

### 2.2. Patients and Data Sources

The study population consisted of courier-related casualties identified from the emergency departments of nine hospitals in İstanbul and Ankara between 1 March 2020, and 1 June 2022, together with linked prehospital and early fatal courier cases verified through official accident, emergency medical service, forensic, police, or hospital records.

Twenty-one patients (2.5%) were injured in Ankara; the remaining 836 (97.5%) accidents occurred in İstanbul. Medical records showed that courier injuries typically occurred on highways and main roads, which traversed the city while the accidents involving pedestrians occurred mainly at pedestrian crossings on inner-city roads.

Clinical data were extracted from emergency department records, orthopedic and trauma consultation records, inpatient files, operative notes, radiological reports, discharge summaries, and follow-up records. Fatal courier cases were not derived solely from the hospital-treated survivor cohort. They were included only when the fatal event could be verified through linked emergency medical service, forensic, police, official accident, or hospital records. These cases were used only for mortality-related and mechanism-specific fatality analyses and were excluded from analyses of surgical treatment, complications, follow-up outcomes, and the primary New Injury Severity Score (NISS) comparison. Therefore, the reported mortality proportion should not be interpreted as the mortality rate of all courier crashes.

### 2.3. Data Collection and Treatment Classification

Data collected included patient age and sex; side and extremity involvement; injury type and severity; presence of fracture, dislocation, or ligament, muscle, tendon, vessel, or nerve injury; associated system injuries; treatment modality; complications; and treatment outcomes. Treatment modality was classified as conservative or surgical. Surgical treatment included fracture fixation, debridement, external fixation, intramedullary nailing, plate or screw fixation, and soft-tissue procedures such as tendon, ligament, wound, vascular, neural, or flap procedures. Conservative treatment included observation, analgesia, wound care, bandaging, casting, bracing, and outpatient follow-up.

### 2.4. Injury Severity Assessment and AIS Coding

Injury severity was assessed using the New Injury Severity Score (NISS). All available clinical, radiological, operative, discharge, emergency medical service, forensic, and official accident records were reviewed for anatomical injury coding. Injuries were coded according to the Abbreviated Injury Scale (AIS) 2005, Update 2008, before calculation of NISS [12]. NISS was then calculated as the sum of the squares of the three highest AIS scores, regardless of body region [13]. The NISS comparison was intended to describe the injury-severity profile of the included hospital-treated clinical groups and was not intended to represent the relative severity of all courier-related crashes in the source population. For the primary group comparison, NISS analysis was restricted to hospital-treated surviving couriers and evaluated or treated pedestrians [14,15].

AIS coding was performed independently by two investigators experienced in trauma data extraction. The investigators were blinded to the comparative study hypotheses and to the final statistical results. Disagreements in AIS coding or NISS calculation were resolved by consensus; when consensus could not be reached, a senior trauma surgeon reviewed the case.

Fatal cases without sufficient anatomical detail were included in mortality analyses but excluded from mean NISS calculations and NISS-based comparisons. Vertebral injuries were classified according to the AO Spine thoracolumbar injury classification system, and neurological status was recorded using the Frankel grading system [16,17].

### 2.5. Courier Injury Mechanism Classification

**Figure 1:**
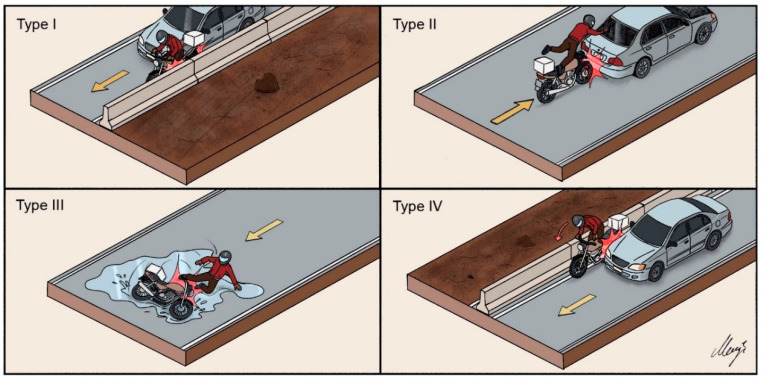
Author-developed four-category descriptive classification of courier injury mechanisms. The illustration was drawn by Muhammed Emin Yorulmaz.

Courier injury mechanisms were categorized using an author-developed four-category descriptive classification based on recurrent crash patterns identified from patient or relative statements and available accident reports. Type I was defined as the courier being crushed between a roadside barrier and a vehicle traveling in the adjacent lane. Type II was defined as the courier striking a stationary or slow-moving vehicle from behind. Type III was defined as slipping or skidding on a wet surface. Type IV was defined as the courier being forced toward a roadside barrier by another vehicle and subsequently falling toward the roadside or ditch (Figure 1). This classification has not been externally validated, has not been used in previous trauma registries, and was not tested for interobserver reproducibility; therefore, mechanism-based analyses were considered exploratory.

### 2.6. Pedestrian Injury Mechanism Classification

Pedestrian injury mechanisms were categorized descriptively based on patient histories and available accident reports. Type A was defined as impact from the tire or fender of the motorcycle. Type B was defined as the motorcycle running over the pedestrian’s foot. Type C was defined as handlebar-related impact. Type D was defined as a fall after being struck by the motorcycle. Pedestrians were grouped by mechanism and compared descriptively in terms of sex, extremity involvement, injury type, and treatment modality.

### 2.7. Statistical Analysis

Statistical analyses were performed using SPSS version 21.0. Continuous variables were assessed for normality using visual inspection of histograms and Q–Q plots and, when appropriate, the Shapiro–Wilk test. Normally distributed continuous variables were presented as mean ± standard deviation, whereas non-normally distributed variables were presented as median and interquartile range or range, as appropriate.

Comparisons between two independent groups were performed using the independent-samples *t*-test for normally distributed variables with comparable variances, Welch’s *t*-test Welch test, or Mann–Whitney U test, as appropriate. Comparisons among more than two mechanism-based subgroups were performed using one-way analysis of variance or Welch’s ANOVA for or the Kruskal–Wallis, as appropriate. Post hoc pairwise comparisons were adjusted using the Holm–Bonferroni method.

Categorical variables were compared using the chi-square test or Fisher’s exact test, as appropriate. Fisher’s exact test was preferred for comparisons involving small expected cell counts or zero-event cells. Continuous and categorical results were reported using outcome-specific denominators. Because the study was retrospective and several comparisons involved structurally zero cells. Results were primarily presented as descriptive statistics with corresponding *p* values. Follow-up-related analyses were restricted to patients with available follow-up data, whereas fatal courier cases were included only in mortality-related and mechanism-specific fatality analyses. Mechanism-based subgroup analyses were considered secondary and exploratory. This study was reported in accordance with the STROBE guidelines for observational studies [18].

As an exploratory age-adjusted analysis, Firth penalized logistic regression was performed because several clinically relevant outcomes involved zero-event cells or complete or near-complete separation between groups. Group status (courier vs. pedestrian) and age were entered as covariates in models of surgical treatment requirement, complications, and mortality. Results were reported as odds ratios (ORs) with 95% confidence intervals using the logistf package in (version 1.26.1) R (version 4.5.1; R Foundation for Statistical Computing, Vienna, Austria) [19,20]. These models were considered exploratory and were not intended to support causal inference because of complete or near-complete separation, zero-event cells, and limited availability of confounding variables.

## 3. Results

### 3.1. Cohort Characteristics

A total of 857 courier-related traffic casualties were identified, including 491 couriers and 366 pedestrians struck by courier-operated motorcycles or mopeds. Among couriers, 111 fatal cases were identified; 71 occurred before hospital admission and were analyzed separately as prehospital deaths, while 40 occurred during transport or within the first 24 h of hospitalization. Clinical treatment and follow-up analyses were restricted to hospital-treated surviving couriers and pedestrians with available clinical records. Because linked fatal-event records were included and minor nonfatal courier injuries may have been underrepresented, this mortality proportion should be interpreted as a mortality proportion within the identified selected dataset, not as the mortality rate of all courier crashes.

The mean age of the injured couriers was 24.4 ± 5.5 years (range: 18–41 years) and the mean follow-up duration among hospital-treated surviving couriers was 32.7 ± 7.5 months (range: 24–46 months). All couriers were male.

Among the 366 pedestrians, the mean age was 37.8 ± 17.6 years. Of these, 180 (49.2%) were female and 186 (50.8%) were male. Upper extremity injuries were observed in 113 (30.9%), whereas 253 patients (69.1%) had lower extremity injuries. Soft tissue injuries or bone edema were present in 275 patients (75.1%), and fractures were identified in 91 (24.9%) (Table 1).

### 3.2. Orthopaedic and Associated Injuries Among Couriers

A total of 111 fatal courier cases were identified. Fatal trauma was associated with severe head injury, intracranial bleeding, thoracoabdominal organ injury, hemopneumothorax, or intra-abdominal hemorrhage. Among the 380 hospital-treated surviving couriers, all had surgically treated musculoskeletal trauma, including multiple fractures, open fractures, complex periarticular injuries, or fracture-associated soft-tissue injuries. Because fracture combinations were highly heterogeneous, detailed fracture-level descriptions were summarized in Supplementary Tables S1–S3, rather than described individually in the main text.

Open comminuted long-bone fractures occurred in 138 patients (36.3%), most frequently involving the tibia, followed by the femur, humerus, and radius/ulna. Among open fractures, Gustilo-Anderson Type I–II injuries were generally treated with intramedullary fixation, whereas Type III injuries commonly required debridement and temporary external fixation [21,22]. Associated non-orthopaedic injuries included head trauma, hemopneumothorax requiring drainage, and abdominal trauma requiring splenectomy. Early or late complications occurred in 53 hospital-treated surviving couriers (13.9%), most commonly delayed wound healing, superficial wound infection, chronic osteomyelitis, pseudoarthrosis or malalignment, and post-traumatic ankle stiffness or arthrosis. Detailed injury distributions and complication types are summarized in Table 2.

### 3.3. Courier Injury Mechanisms

Patients were grouped into four author-developed mechanism categories. Mechanism groups differed significantly in age, with the lowest mean age observed in Type IV couriers (*p* = 0.0004). Follow-up duration did not differ significantly among groups. Type I, Type II, and Type IV injuries showed similar high-energy patterns, whereas Type III sliding or skidding injuries showed a significantly higher proportion of single-fracture injuries with associated soft-tissue injury (48.3%; *p* < 0.0001). Mean NISS values were similar among Type I, Type II, and Type IV injuries, while Type III injuries had a significantly lower mean NISS (29.9 ± 4.9; *p* < 0.0001) (Table 3).

### 3.4. Pedestrian Injuries and Mechanisms

No significant differences were found between male and female pedestrians regarding age, follow-up duration, extremity involvement, injury type, or treatment modality. Soft-tissue trauma occurred in 275 pedestrians (75.1%), and fractures occurred in 91 pedestrians (24.9%). Overall, 106 pedestrians (29%) underwent surgical intervention, including fracture fixation and soft-tissue procedures; the remaining 260 pedestrians (71%) were treated conservatively with bandaging, casting, bracing, analgesia, and outpatient follow-up.

Pedestrian mechanisms differed significantly in terms of upper versus lower extremity involvement, soft-tissue versus fracture pattern, and treatment modality. Type A and Type B injuries mainly involved the lower extremity, Type C injuries involved the upper extremity, and Type D injuries had the highest fracture proportion (45.5%) (Table 4).

### 3.5. Comparison Between Couriers and Pedestrians

Significant differences were observed between groups in terms of age, with couriers being younger (*p* < 0.001). Multiple fractures, open fractures, high NISS values, complications, mortality, and surgical treatment were more frequent among couriers than pedestrians. However, this comparison should be interpreted descriptively because the courier and pedestrian cohorts were identified through different clinical and linked-record pathways and represented different injury-severity spectra.

All hospital-treated surviving couriers included in the orthopedic trauma cohort underwent surgery, whereas most pedestrians were treated conservatively (71%; *p* < 0.0001). This finding should be interpreted in the context of the case-identification process, which preferentially captured severe musculoskeletal trauma cases.

Age-adjusted Firth penalized logistic regression was performed as an exploratory analysis because several outcomes showed complete or near-complete separation between groups. Compared with pedestrians, couriers showed higher estimated odds of surgical treatment requirement, complications, and mortality (Table 5). However, these models should be interpreted as exploratory and descriptive because of complete or near-complete separation, zero-event cells, and very wide confidence intervals. Therefore, the regression results should not be interpreted as evidence of independent causal effects.

## 4. Discussion

The principal finding of this multicenter retrospective study was that, within a selected hospital-based cohort, courier casualties identified through hospital, trauma referral, emergency, forensic, and linked fatal-event records showed a more severe clinical profile than pedestrians evaluated after being struck by courier-operated motorcycles or mopeds. However, this finding must be interpreted in the context of substantial selection, referral, and ascertainment bias. The courier cohort was enriched for severe musculoskeletal trauma and linked fatal cases, whereas the pedestrian cohort included a broader spectrum of mostly minor injuries.

The study period coincided with the COVID-19 pandemic, during which delivery services became more visible in urban traffic and motorcycle accident rates were reported to increase in Turkey [23,24]. However, because the present study did not include pre-pandemic or post-pandemic comparison groups, temporal trend analysis, or exposure data, the effect of the pandemic on courier-related injuries could not be directly evaluated.

Beyond the pandemic context, previous studies have identified several potentially relevant factors in motorcycle and food-delivery rider crashes, including helmet-use behavior, hazardous traffic scenarios, delivery workload, and occupational and behavioral characteristics [25,26,27,28]. These factors were not consistently available in the present retrospective dataset and therefore could not be evaluated as potential confounders or causal determinants of injury severity.

Urban pedestrian studies have also emphasized the importance of the traffic environment and targeted safety measures, particularly for vulnerable pedestrian groups [29]. However, because the present study lacked population-level exposure and detailed environmental data, these findings provide contextual support rather than directly comparable evidence.

The mortality proportion observed in the present selected courier dataset was higher than that reported in most general motorcycle trauma cohorts and trauma registry studies. Granieri et al. analyzed 1725 consecutive motorcycle trauma patients and reported an overall mortality rate of 4.9% [30]. Eden et al., using the TraumaRegister DGU, evaluated critically injured motorcyclists and reported increased mortality among patients older than 65 years [31]. Hsieh et al. analyzed motorcycle-related hospitalizations in a trauma registry system in southern Taiwan and emphasized the influence of age, injury severity, and injury pattern on outcomes [32]. Compared with these studies, the higher mortality proportion in the present cohort is likely explained by the inclusion of linked prehospital and early fatal courier cases and by the underrepresentation of minor nonfatal courier injuries. Therefore, the mortality figure in the present study should be compared with caution and should not be interpreted as a generalizable fatality rate for all courier-related motorcycle crashes.

The marked difference in NISS between groups also requires cautious interpretation. Among hospital-treated surviving couriers, the mean NISS was 39.2 ± 8.2, whereas the mean NISS among pedestrians was 2.4 ± 1.6. Such an extreme difference indicates that the two groups represented fundamentally different clinical populations with largely non-overlapping injury-severity spectra; therefore, the clinical interpretability of a formal statistical comparison between them is limited. Nevertheless, NISS values are reported to transparently characterize the injury-severity profiles of each group as identified through their respective case-ascertainment pathways. These values should not be interpreted as population-level estimates of relative injury severity between couriers and pedestrians, or as evidence that courier injuries are inherently more severe than pedestrian injuries across all crash scenarios.

Similarly, the finding that all hospital-treated surviving couriers underwent surgery should not be generalized to all courier-related injuries. In routine trauma practice, some motorcycle crash patients would be expected to sustain injuries suitable for nonoperative treatment. In the present study, the surgical rate reflects the orthopedic trauma-based case-identification process and the referral nature of the participating centers, which preferentially captured severe musculoskeletal trauma cases.

The age-adjusted Firth regression analyses were performed only as exploratory descriptive analyses. Although the estimated odds ratios were high, the models were affected by complete or near-complete separation, zero-event cells, and very wide confidence intervals. In addition, several important confounding variables, including helmet use, vehicle speed, road conditions, traffic density, alcohol use, protective equipment, time of injury, work intensity, and driving experience, were unavailable. Therefore, these adjusted estimates should not be interpreted as evidence that group status independently or causally explains the observed differences in clinical outcomes.

Mechanism-based findings suggested that Type I, Type II, and Type IV courier injuries had higher-energy clinical profiles than Type III sliding or skidding injuries. However, because the mechanism classification was author-developed and has not been externally validated or tested for interobserver reproducibility, these mechanism-based findings should be interpreted as exploratory and hypothesis-generating. The mechanism categories may nevertheless provide a clinically understandable framework for describing recurrent crash scenarios in the present dataset.

Among pedestrians, most injuries were low-severity soft-tissue injuries or bone edema, and most patients were treated conservatively. Type D injuries, corresponding to falls after being struck, showed the highest fracture proportion. These findings suggest that pedestrians struck by courier-operated motorcycles or mopeds may present with a broad spectrum of injury severity, but the present data do not permit estimation of the true incidence of pedestrian injuries or comparison of risk between pedestrians and couriers.

The major limitation of this study is selection bias affecting the primary comparison. The courier cohort was derived mainly from trauma referral pathways and consisted of hospital-evaluated courier casualties with documented musculoskeletal trauma; therefore, minor courier injuries that did not require orthopedic evaluation or hospital-level treatment may have been underrepresented. In contrast, the pedestrian cohort included a broader spectrum of injuries ranging from minor soft-tissue trauma to fractures. Consequently, the observed differences in injury severity, surgical treatment rates, complications, and mortality may partly reflect differences in case selection rather than true population-level differences between couriers and pedestrians.

Additional limitations include the retrospective design, potential referral bias related to the participating trauma centers, possible ascertainment bias from linked fatal-event records, and absence of exposure data such as number of deliveries, distance traveled, hours worked, accident incidence, traffic density, weather conditions, road design, helmet use, protective equipment, vehicle speed, alcohol or drug use, and traffic-rule violations. The study also did not include pre-pandemic or post-pandemic comparison groups and therefore cannot determine the actual effect of the COVID-19 pandemic on courier-related injuries. Finally, the mechanism classification was developed for descriptive purposes and requires external validation.

Although the observed mechanisms suggest potential areas for prevention, the present study did not directly evaluate causal risk factors such as speed, helmet use, road conditions, work intensity, traffic violations, or protective equipment. Therefore, any preventive implications should be considered hypothesis-generating rather than evidence of the effectiveness of specific policy interventions.

## 5. Conclusions

In this selected multicenter hospital-based cohort, courier casualties identified through hospital, trauma referral, emergency, forensic, and linked fatal-event records demonstrated a more severe clinical profile than pedestrians evaluated after being struck by courier-operated motorcycles or mopeds. However, this difference should be interpreted in light of substantial selection, referral, and ascertainment bias. The courier cohort was enriched for severe musculoskeletal trauma and linked fatal cases, whereas the pedestrian cohort included a broader spectrum of mostly minor injuries. Therefore, the present findings should not be interpreted as population-level estimates of accident incidence, relative injury risk, or public health burden. Future population-based studies incorporating exposure data, crash circumstances, helmet use, vehicle speed, road conditions, work intensity, and occupational factors are required.

## Figures and Tables

**Table 1:** Demographic characteristics and main outcome comparison between courier and pedestrian casualty groups, with fatal courier cases analyzed separately.

Variable	Couriers	Pedestrians Struck by Couriers	*p* Value
Total patients	491 (57.3%)	366 (42.7%)	-
Age, years	24.4 ± 5.5 (18–41)	37.8 ± 17.6 (14–75)	<0.001
Sex	Male: 491 (100%); female: 0	Male: 186 (50.8%); female: 180 (49.2%)	-
Follow-up, months	32.7 (24–46)	32.4 (24–43)	-
Extremity involvement	Upper and lower extremities	Upper: 113 (30.9%); lower: 253 (69.1%)	-
Soft tissue injury only	0	275 (75.1%)	<0.0001
Fracture among evaluated/treated patients	380/380 (100%)	91/366 (24.9%)	<0.0001
Open fractures	138/380 (36.3%)	0	<0.0001
Mortality	111/491 (22.6%) *	0	<0.0001
NISS	39.2 ± 8.2	2.4 ± 1.6	<0.0001
Conservative treatment	0	260/366 (71%)	<0.0001
Surgical treatment	380/380 (100%)	106/366 (29%)	<0.0001
Complications	53/380 (13.9%)	0	<0.0001

* The mortality proportion of 22.6% includes prehospital and early fatal courier cases verified through emergency medical service, forensic, police, and hospital records. Because minor nonfatal courier injuries may have been underrepresented in the source dataset, this figure reflects a mortality proportion within the identified selected cohort and should not be interpreted as the case-fatality rate of all courier-related motorcycle crashes or as a population-level mortality rate.

**Table 2:** Selected orthopaedic and associated injury patterns with clinical relevance.

Category	Finding	Number/Proportion	Treatment or Clinical Note
Courier long-bone fractures	Tibia	70/138 (50.7%)	Most frequent long-bone fracture site
Courier long-bone fractures	Femur	39/138 (28.3%)	-
Courier long-bone fractures	Humerus	19/138 (13.7%)	-
Courier long-bone fractures	Radius/ulna	10/138 (7.3%)	-
Open-fracture management	Gustilo-Anderson Type I–II	74/138 (53.6%)	Closed reduction and intramedullary nailing
Open-fracture management	Gustilo-Anderson Type III	64/138 (46.4%)	Debridement and temporary external fixation
Courier associated injuries	Head trauma	147/380 (38.6%)	33 required neurosurgical intervention
Courier associated injuries	Hemopneumothorax	61/380 (16.1%)	Required underwater drainage
Courier associated injuries	Splenectomy	31/380 (8.2%)	Performed for abdominal trauma
Courier vertebral injuries	Any vertebral injury	11/380 (2.9%)	Spectrum from sprain/transverse-process fracture to AO Type C injury
Courier complications	Any early or late complication	53/380 (13.9%)	Most frequent: delayed wound healing and infection
Pedestrian soft-tissue injuries	Any soft-tissue injury	275/366 (75.1%)	Most treated conservatively with recovery
Pedestrian fractures	Any fracture	91/366 (24.9%)	74 fractures reportedly required surgical fixation
Pedestrian fractures	Metatarsal fractures	12/366 (3.3%) patients	Most common fracture group reported
Pedestrian fractures	Femoral neck fractures	10/366 (2.7%) patients	-
Pedestrian fractures	Tibial diaphysis fractures	9/366 (2.5%) patients	-

**Table 3:** Injury mechanism and outcome distribution among courier casualties, with survivor-based outcomes calculated among hospital-treated surviving couriers and fatal cases reported separately.

Variable	Type I (*n* = 115)	Type II (*n* = 103)	Type III (*n* = 60)	Type IV (*n* = 102)	Total (*n* = 380)	*p* Value
Age, years	24.9 (18–39)	25.1 (18–40)	25.6 (18–41)	22.4 (18–36)	24.4 (18–41)	0.0004
Follow-up, months	31.9 (24–44)	32.1 (24–45)	34.2 (24–46)	33.4 (24–46)	32.7 (24–46)	0.160
Multiple fractures + soft tissue injury	115/115 (100%)	103/103 (100%)	31/60 (51.7%)	102/102 (100%)	351/380 (92.4%)	<0.0001
Single fracture + soft tissue injury	0	0	29/60 (48.3%)	0	29/380 (7.6%)	<0.0001
Deaths reported by mechanism	40	41	0	30	111	-
NISS	40.9 ± 7.6	41.0 ± 7.7	29.9 ± 4.9	40.8 ± 7.1	39.2 ± 8.2	<0.0001
Surgical treatment	115/115 (100%)	103/103 (100%)	60/60 (100%)	102/102 (100%)	380/380 (100%)	-

**Table 4:** Injury mechanism and outcome distribution among pedestrians struck by courier-operated motorcycles or mopeds.

Variable	Type A (*n* = 168)	Type B (*n* = 66)	Type C (*n* = 66)	Type D (*n* = 66)	Total (*n* = 366)	*p* Value
Follow-up, months	32.7 (24–43)	32.9 (24–42)	33.2 (27–41)	30.3 (24–40)	32.4 (24–43)	0.997
Male	83 (49.4%)	34 (51.5%)	35 (53.0%)	34 (51.5%)	186 (50.8%)	0.996
Female	85 (50.6%)	32 (48.5%)	31 (47.0%)	32 (48.5%)	180 (49.2%)	-
Upper extremity injury	0	0	66/66 (100%)	47/66 (71.2%)	113 (30.9%)	<0.0001
Lower extremity injury	168/168 (100%)	66/66 (100%)	0	19/66 (28.8%)	253 (69.1%)	-
Soft tissue injury	135/168 (80.4%)	52/66 (78.8%)	52/66 (78.8%)	36/66 (54.5%)	275 (75.1%)	<0.001
Fracture	33/168 (19.6%)	14/66 (21.2%)	14/66 (21.2%)	30/66 (45.5%)	91 (24.9%)	-
Conservative treatment	113/168 (67.3%)	56/66 (84.8%)	58/66 (87.9%)	33/66 (50.0%)	260 (71%)	<0.001
Surgical treatment	55/168 (32.7%)	10/66 (15.2%)	8/66 (12.1%)	33/66 (50.0%)	106 (29%)	-

Mechanisms: Type A = struck by tire/fender; Type B = foot run-over; Type C = handlebar impact; Type D = fall after being struck.

**Table 5:** Exploratory age-adjusted Firth penalized logistic regression models.

Outcome	Adjusted OR	95% CI	*p* Value	Interpretation Note
Surgical treatment requirement	1666	102–27,162	<0.0001	Complete or near-complete separation
Complications	75.7	5.2–1110	0.002	Zero-event cells in pedestrian group
Mortality	181.8	11.7–2837	<0.0001	Includes linked fatal courier cases

## Data Availability

The data supporting the findings of this study are not publicly available due to patient confidentiality, institutional restrictions, and ethical approval requirements. Anonymized data may be made available from the corresponding author upon reasonable request and with the approval of the Istanbul Faculty of Medicine Clinical Research Ethics Committee and the participating institutions. Level of evidence: Level III, retrospective cohort study.

## Supplementary Materials

The following supporting information can be downloaded at: https://www.mdpi.com/article/10.3390/jcm15145383/s1, Table S1: Detailed fracture distribution among hospital-treated surviving couriers. Table S2: Vertebral injuries among couriers according to AO Spine classification and Frankel grade. Table S3: Early and late complications among hospital-treated surviving couriers. Table S4: Case identification and analytic cohorts.
