# Supplementary material for: Hospital-Based Injury Patterns Among Motorcycle Couriers and Pedestrians Struck by Courier-Operated Motorcycles and Mopeds: A Multicenter Retrospective Study
Source: J Clin Med. 2026 Jul 9;15(14):5383. doi: 10.3390/jcm15145383 (PMC13411095; doi:10.3390/jcm15145383)
Supplement: Supplementary file 1 [file jcm-15-05383-s001.zip › jcm-4391689-supplementary.pdf]

Supplementary Tables

Table S1. Detailed fracture distribution among hospital-treated surviving couriers.

Injury category	Specific injury	n	% of relevant denominator	Additional note
**Open comminuted long-bone fracture**	Tibia	70	50.7% of open long-bone fractures; 18.4% of hospital-treated surviving couriers	Most frequent long-bone fracture site
**Open comminuted long-bone fracture**	Femur	39	28.3% of open long-bone fractures; 10.3% of hospital-treated surviving couriers	—
**Open comminuted long-bone fracture**	Humerus	19	13.7% of open long-bone fractures; 5.0% of hospital-treated surviving couriers	—
**Open comminuted long-bone fracture**	Radius/ulna	10	7.3% of open long-bone fractures; 2.6% of hospital-treated surviving couriers	—
**Open-fracture management**	Gustilo-Anderson Type I-II	74	53.6% of open long-bone fractures	Generally treated with closed reduction and intramedullary fixation
**Open-fracture management**	Gustilo-Anderson Type III	64	46.4% of open long-bone fractures	Generally required debridement and temporary external fixation
**Closed complex fracture**	Acetabular fracture	9	3.7% of closed-fracture subgroup; 2.4% of hospital-treated surviving couriers	—
**Closed complex fracture**	Pilon fracture	7	2.9% of closed-fracture subgroup; 1.8% of hospital-treated surviving couriers	—
**Closed complex fracture**	Bimalleolar fracture	11	4.5% of closed-fracture subgroup; 2.9% of hospital-treated surviving couriers	—
**Closed complex fracture**	Humeral diaphysis fracture	7	2.9% of closed-fracture subgroup; 1.8% of hospital-treated surviving couriers	—
**Closed complex fracture**	Distal humerus fracture	6	2.5% of closed-fracture subgroup; 1.6% of hospital-treated surviving couriers	—
**Closed complex fracture**	Distal radius fracture	11	4.5% of closed-fracture subgroup; 2.9% of hospital-treated surviving couriers	—
**Closed complex fracture / dislocation**	Shoulder dislocation	3	1.2% of closed-fracture subgroup; 0.8% of hospital-treated surviving couriers	—
**Closed complex fracture**	Clavicle fracture	3	1.2% of closed-fracture subgroup; 0.8% of hospital-treated surviving couriers	—
**Closed complex fracture-dislocation**	Elbow fracture-dislocation	3	1.2% of closed-fracture subgroup; 0.8% of hospital-treated surviving couriers	—
**Single fracture + associated soft-tissue injury**	Overall subgroup	29	7.6% of hospital-treated surviving couriers	Includes ligament sprains, ACL ruptures, intramuscular hematomas, and rotator cuff injuries

Table S2. Vertebral injuries among couriers according to AO Spine classification and Frankel grade.

Vertebral injury type	n	Rate among vertebral injuries	Frankel after injury	Postoperative Frankel	Treatment	Final Frankel
**Paravertebral muscle sprain**	3	27.3%	E	E	Conservative	E
**Two-level transverse-process fracture on the same side (L3-L4 and L4-L5)**	2	18.2%	E	E	Conservative	E
**AO Type A1 at T12**	1	9.1%	E	E	Conservative	E
**AO Type A3 at T12 and L1**	2	18.2%	E	E	Posterior instrumentation	E
**AO Type B at L1**	1	9.1%	D	E	Posterior instrumentation	E
**AO Type C at T11-T12**	1	9.1%	A	A	Posterior instrumentation	A
**AO Type C at L5-S1**	1	9.1%	D	E	Posterior instrumentation	E

Table S3. Early and late complications among hospital-treated surviving couriers.

Timing	Complication	n	% of complications	Clinical note
**Early**	Pulmonary embolism	1	1.9%	—
**Early**	Atelectasis	3	5.7%	—
**Early**	Deep vein thrombosis	4	7.5%	—
**Late**	Delayed/impaired wound healing	15	28.4%	Most frequent complication
**Late**	Superficial wound infection	11	20.8%	Reported as Staphylococcus aureus infections treated with parenteral antibiotics, debridement, and secondary closure
**Late**	Chronic osteomyelitis	4	7.5%	—
**Late**	Pseudoarthrosis/malalignment	9	16.9%	Reported in 5 open tibia fractures and 4 open femoral diaphysis fractures
**Late**	Bed sore	1	1.9%	Observed in one paraplegic patient with vertebral fracture; local flap closure
**Late**	Traumatic ankle arthrosis / loss of range of motion	5	9.4%	Observed in pilon fracture patients
**Total**	Any early or late complication	53	100%	13.9% of hospital-treated surviving couriers

Table S4. Case identification and analytic cohorts.

Cohort component	n	Data source	Clinical treatment/follow-up analyses	Mortality analyses	Primary NISS comparison
**Total courier-related traffic casualties**	857	Hospital records and linked fatal-event records	Variable by subgroup	Yes, where applicable	No, not as a total cohort
**Couriers, total**	491	Hospital records plus linked prehospital/early fatal-event records	Only hospital-treated surviving couriers included	Yes	Partly
**Hospital-treated surviving couriers**	380	Emergency, orthopedic/trauma consultation, inpatient, operative, radiology, discharge, and follow-up records	Included	Included as survivors	Included
**Linked fatal courier cases**	111	Emergency medical service, forensic, police, official accident, or hospital records	Excluded	Included	Excluded
**Pedestrians struck by courier-operated motorcycles/mopeds**	366	Hospital records	Included	Included as no deaths identified in dataset	Included
